# Primary pure large cell neuroendocrine carcinoma of the ovary: A rare case report and review of literature

**DOI:** 10.1097/MD.0000000000022474

**Published:** 2020-12-04

**Authors:** Xue Peng, Hongjing Wang

**Affiliations:** Department of Obstetrics and Gynecology, West China Second Key Laboratory of Birth Defects and Related Diseases of Women and Children (Sichuan University), Ministry of Education, West China Second University Hospital, Sichuan University, Chengdu, Sichuan, P.R. China.

**Keywords:** clinical characteristics, large cell neuroendocrine carcinoma, ovary, pathology, prognosis

## Abstract

**Introduction:**

Ovarian large cell neuroendocrine carcinoma (LCNEC), or ovarian non-small cell neuroendocrine carcinoma, which is a newly described tumour in the classification of primary ovarian neoplasms by the World Health Organization, is a rare entity that is frequently associated with a surface epithelial and germ cell neoplasm component. Few cases have been reported in the literature, and only 18 primary pure ovarian LCNEC cases have been reported so far, including our 1 case. Ovarian LCNEC is a highly aggressive tumor with a poor prognosis even at an early stage.

**Patient concerns:**

We report a case of a 55-year-old postmenopausal woman who complained of abdominal pain. CT examination revealed a mass in the right adnexial region and CA125 level was elevated.

**Diagnosis:**

She underwent a exploratory laparotomy, and diagnosed as LCNEC histopathologically.

**Interventions:**

Cytoreductive surgery was administered to the patient, and had accepted 5 cycles of chemotherapy consisting of paclitaxel and cisplatin.

**Outcomes:**

Follow-up for 12 months showed no clinical or radiological evidence of disease recurrence.

**Conclusion:**

This case is 1 of the ovarian LCNEC which is a rare and extremely malignant tumor. Diagnosis requires histopathology and immunohistochemistry. The treatment includes primary cytoreductive surgery followed by chemotherapy.

## 1 Introduction

Neuroendocrine tumors are a group of tumors that originate from amine precursor uptake and decarboxylation (APUD) cells, which generally occurs in the digestive system and lung. Primary and secondary gynecologic neuroendocrine tumors are both uncommon, accounting for only 2% of gynecologic tumors.^[1]^ Ovarian large cell neuroendocrine carcinoma (LCNEC) is synonymous with ovarian NSCNE,^[2]^ which is a newly described tumour in the classification of primary ovarian neoplasms according to the World Health Organization (2003).^[3]^ It can be divided into 2 types, one is pure which only contains LCNEC, another is mixed which compose with surface epithelial or germ cell neoplasm. Since Collins^[4]^ reported the first case of ovarian LCNEC in 1991, only a total of 60 cases have been reported in the literature so far, including our 1 case, and all were case reports. Moreover literature reports is mixed more, pure only 17 cases, ours for 18 cases.

Ovarian LCNEC is a highly aggressive tumor with a poor prognosis even at an early stage. Due to the rarity of the disease and the lack of systematic population-based studies or registration data, there is hitherto no consensus on the optimal treatment.

We report a case of recent encounter of pure ovarian LCNEC, and summarize the clinicopathological features with a literature, so as to deepen the recognition of this disease.

## 2 Case presentation

A 55 year old, gravida 2, parity 1, postmenopausal woman complained of abdominal pain, and used of antibiotics but not effective self-intermittent a month ago, she had no chest tightness, cough, hemoptysis, hematochezia and other symptoms. A CT examination revealed that a irregular, poorly circumscribed, cystic and solid mass was felt occupying the right adnexial region measuring 7 × 6 × 6 cm with ovarian vascular pedicle sign. In addition, multiple pelvic lymph node enlargement was shown. Her CA125 level was elevated to 443.6*U/ml*, and Pap cervical cancer screening was normal. Gastroscope and chest X-ray were within normal limits. A exploratory laparotomy was carried out and 200 *ml* of bloody ascitic fluid was drained. Laparotomy demonstrated an irregular and solid mass of the right ovary measuring 8 × 7 × 5 cm, which was dense adhesion with sigmoid colon, rectum, appendix and parietal pelvic wall. Scattered miliary nodules are seen in the abdominal cavity measuring 0.1–0.2 *cm* in diameter. Multiple right pelvic lymph nodes and para-aortic lymph nodes were enlarged about 2 cm in diameter, and prostrate beside the abdominal aorta and vena cava. A right salpingo-oophorectomy was performed, and intraoperative frozen section showed poorly differentiated carcinoma of the right ovary. Consquently, a total abdominal hysterectomy, bilateral salpingo-oophorectomy, pelvic lymphadenectomy, omentectomy, and appendectomy were performed.

### 2.1 Pathology

The samples displayed a right ovarian tumor measuring 8 × 7 × 5 *cm*, capsule intact, external surface was irregular and nodular, and its cut section shows a solid, a yellow-white focus and partial old hemorrhage and a massive necrotic area.

### 2.2 Microscopy

Poorly differentiated malignant tumour-carcinoma were shown in the right ovary. Right fallopian tube, uterine wall and right pelvic lymph nodes showed tumour deposits. When the hematoxylin and eosin stained slides were examined under the microscope, the tumour comprised mostly of solid sheets, islands, or bands formations, less tumor stroma, accompanied by wide areas of necrosis. It had large round to ovoid nuclei, occasionally with prominent nucleoli, coarse chromatin, and frequently showed a high mitotic rate.

### 2.3 Immunohistochemistry (IHC)

IHC was performed in order to confirm the ultimate histological diagnosis. The tumor cells were positive for P53, P16, CD56, neuron-specific enolase (NSE), with focal staining for cytokeratins (CK)-P. While immunostaining for synaptophysin, WT-1, CK7, CK20, ER, PR, and chromogranin A were negative. Ki67 positive rate is about 85%.

According to the clinical manifestation, histopathological features, and IHC profiles, suggested a final diagnosis of ovarian LCNEC as International Federation of Gynecology and Obstetrics clinical stage III.

The patient recovered well postoperatively, and blood examination of NSE and CA125 gradually decreased (Table 1).

**Table 1:** Tumor marks shown in our case of ovary LCNEC.

Stage	CA125 U/mL (normal<35)	CA199 U/mL (normal<30.9)	HE4 pmol/l (normal premenopausal<=70, postmenopausal<=140)	NSE ng/mL (normal<15)
preoperative	443.6	—	—	—
before 1st cc;po	175.1/75.9	3.7/5.3	29.8	44.85
before 2ed cc;po	13.2	9.7	30.7	17.34
before 3rd cc;po	—	—	—	12.21
Latest results (po 11 mo)	4.2	7.5	—	14.32

“cc” = cycles of chemotherapy, “po” = postoperative, “-” = this check is not performed, LCNEC = large cell neuroendocrine carcinoma.

Five cycles of postoperative paclitaxel and carboplatin chemotherapy were completed 8 months ago.

Simultaneously, it is recommended the patient with radiation consulting, but did not receive radiotherapy yet. Eleven months after the operation, right inguinal lymph node enlargement was found, with a maximum of 2.8 × 1.1cm, however, biopsy indicated that it was only lymphatic tissue and adipose fibrous tissue, and tumor markers were also within the normal range.Follow-up for 12 months showed no clinical or radiological evidence of disease recurrence now.

## 3 Discussion

Neuroendocrine tumors originate from APUD cells, which generally occurs in the digestive system and lung, and also present in normal epithelium of the female genital tract.^[5]^ Several theories have been proposed as to the origin of ovarian LCNEC, including just overgrown from the APUD cells present in epithelial tumors or teratomas,^[2]^ the potential of primitive endodermal cells to differentiate into other cell types.^[4]^ The other possibility is that ovarian LCNEC may develop from non-neuroendocrine cells through activation of genes promoting neuroendocrine differentiation.^[6]^ Ovarian LCNEC is a very rare tumour. To the best of our knowledge, only a total of 60 cases have been reported in the literature, including our 1 case. 42 cases were mixed while 18 cases were pure (Table 2). The present case is the eighteenth case of pure ovarian LCNEC.

**Table 2:** Clinicopathologic,treatment modality, tumor markers, and follow-up of reported cases with pure ovarian LCNEC.

Case (No.)	Authors	Age	Size Dimension, Laterality, gross findings	Stage (FIGO)	clinical manifestation	Tumor markers	Primary operation	Metastases at primary operation	Post-operative therapy (cycles)	Follow-up (time after operation )	complications
1	Behnam et al, 2004^[7]^	27	17 cm, Left, solid and cysts	Ia	pelvic mass	normal	Resection of ovarian tumor;OMT;APP;PLD;right OB;PB	None	TC (6)	NRM (10 m)	None
2	Yasuda et al, 2006^[8]^	44	9 cm, Right, solid	IIIc	abdominal distension	CA125 1200 U/ml	TAH;BSO;OMT	unknow	chemotherapy (details unknown)	DOD (20m)	None
3	Lindboe, 2007^[9]^	64	14c, Right, solid	Ia	abdominal discomfort and nausea	CA125 380U/ml (normal <35); CEA 36mg/L (normal <5)	TAH;BSO;OMT	None	BEP	NRM (9 m)	Breast carcinoma
4	Veras et al, 2007^[2]^	42	Laterality and size unknow, cysts	IV	Pelvic pain	unknow	TAH;BSO	unknow	cisplatinum-based chemotherapy (at least 6)	DOD (20 m)	unknow
5	Dundr et al, 2008^[10]^	73	9 cm, Left, solid	IV	metastatic CNS diseas, dysartria and difficulties in verbal expression	NSE 23mg/l;CA125 94kU/ml; CA199 133U/ml	TAH;BSO;OMT;left-sided nephrectomy;mesenterial metastasis resection	Mesenterium; left renal capsule;CNS	TC	CNS recurrence γ knife (2 m) NRM (12 m)	endometrialcarcinoma, breast carcinoma
6	Tsuji et al, 2008^[11]^	46	15 cm, Right, solid	III	abdominal distension	CA125 914U/mL;LDH 1790IU/L (normal<40));NSE 210U/mL (<10)	TAH;BSO;OMT	Omentum; peritoneum uterine corpus	TC	DOD (4 m)	None
7	Aslam et al, 2009^[12]^	76	35 cm, Left, solid and cysts	IIb	abdominal pain	normal	TAH;BSO;OMT;APP;PL;PAL	None	No	septic shock and died soon before chemotherapy	hypertension
8	Oshita et al, 2011^[13]^	66	11 cm, Right, solid and cysts	IV	multiple lung nodules in a chest X-ray,pelvic mass	CA125 6595 U/ml	TAH;BSO;OMT;PB (TC with 4 sessions before operation)	vagina;lung	TC;wholebrain radiation therapy	Brain metastasis,wholebrain radiation therapy (17 m) NRM (64 m)	None
9	Shakuntala et al, 2012^[14]^	40	7 cm left, 15 cm right, solid and cysts	IIIc	acute distension and pain abdomen;fever, itching	CA125 280.8U/ml; CEA 7.66mg/L	BSO;PAL;OMT;Bladder and sigmoid colon deposit excision (TAH;PL because of CINIII 9 m ago)	Bladder; sigmoid colon	EP (5)	NRM (6 m)	None
10	Lin et al, 2014^[15]^	50	25 cm, Left, solid	IV	pelvic mass	CA125 685.8U/mL	TAH;BSO;partial OMT;APP	liver	TC (3)	pulmonary metastases after 3 sessions of chemotherapy, died 3 m sustaining intracranial hemorrhage following an accidental fall-down injury.	multiple myomas and adenomyosis
11	Ki EY et al, 2014^[16]^	77	15 cm, Unknow, solid	IV	abdominal distension	CA125 124 U/ml	TAH;pelvic mass;neck masses	neck LN	EP	septic shock and died 1.5 m	coronary artery disease
12		58	Unknow, Left, unknow	Ia	abdominal discomfort	unknow	First:TAH;BSO;OMT;PL second:left PAL	First:none second:left para-aortic lymph node	First:TC (6) second:taxoter (7)	recurrence 9 m multiple organ failure and died 17 m	None
13		67	13 cm, Left, solid and cysts	III	urinary frequency	CA125 71.8 U/ml	TAH;BSO;OMT;PL;PAL; multiple biopsy	pelvic peritoneum	TC	NRM (5 m)	None
14	Agarwal et al , 2016^[17]^	35	6 cm, Left, solid and cysts	IIIc	Abdominal pain, amenorrhea; vaginal bleeding	CA125 and CEA were raised	TAH,;BSO	Cervix; LN	No	AWD (3 m)	None
15	Herold et al, 2018^[18]^	75	13 cm, Right, solid	IV	unknow	CA125 63.4U/ml	BSO;OMT;PL;PAL;peritonea;liver (TAH,APP at the age of 37)	liver	EP (2)+TC (2)	NER (36 m)	breast cancer
16	Doğanay et al, 2019^[19]^	73	10 cm, Right, solid	unknow	pelvic pain	normal	TAH;BSO	unknow	EP (6)	NRM (4 m)	None
17	Yang X et al, 2019^[20]^	70	20 cm, Right, solid and cysts	IIIc	abdominal distension	CA125 367.90 U/ml; NSE 24.83 ng/ml	TAH;BSO;OMT;pelvic metastases	surface of the oviduct;partial perimetrium; pelvic area	EP (3)	NRM (3 m)	None
18	Present case	55	8 cm, Right, solid	III	abdominal pain	CA125 443.6U/ml	TAH;BSO;OMT;APP;PL	right fallopian tube;uterine wall,right LN	TC (3)	NER (3m)	

APP = Appendectomy, AWD = alive with disease, BEP = bleomycin, cis-platin and etoposide, BSO = bilateral salpingooophorectomy, DOD = dead of disease, EP = etoposide and cisplatin, FIGO = International Federation of Gynecology and Obstetrics LN = lymph nodes, LSO = Left salpingooophorectomy, M = month(s), NRM = no recurrence or metastases, OB = ovary biopsy, OMT = Omentectomy, PAL = para-aortic lymph node dissection, PB = peritoneal biopsy, PL = Pelvic lymphadenectomy, RSO = right salpingooophorectomy, TAH = total hysterectomy, TC = paclitaxel and carboplatin.

It has been reported in the literature that ovarian LCNEC can be developed in premenopausal and postmenopausal women, ranging from 18 to 80 years, while 6–8 years younger than other common types of epithelial ovarian cancers. The age of pure type was ranging between 27 to 77 years and a median age of 41 years. Clinical manifestation is often similar to the presentation of epithelial ovarian cancer at initial presentation. The most common clinical manifestation was abdominal pain and distension in 66.7%(12/18) of cases, similarly to our case. Followed by pelvic mass (3/18), other clinical symptoms were amenorrhea and vaginal bleeding (1/18), dysphonia and difficulties because of the metastasis to the CNS (1/18)and urinary frequency (1/18). One side was more common and no significant difference between left and right, only 2 cases were reported in bilateral ovaries,^[14,21]^ and 1 case was microscopically involved in contralateral ovaries.^[13]^ Most of the LCNEC are partially solid or cystic, with size ranging from 6 and 35cm (mean size, 14.53 ± 6.36 cm). In present case was a solid mass with size of 8 cm. The CA125 levels range from 63.4 to 6595*U/ml*. Eventhough the level of CA125 usually increase in epithelial ovarian cancers and correlate with cancer stages or responses to treatment, Ki EY et al^[16]^ have reported that the CA125 levels are not specific to clinical courses in ovarian LCNEC. Other tumor markers including CEA, NAE, and so on may rise but not specific. Ovarian LCNEC metastasis in the pelvis and peritoneal cavity is common, while lung, brain and bone are relatively rare.^[10,22]^ Cokmert et al^[23]^ first reported a case of ovarian LCNEC skin metastasis in the extremities 2 months after operation.

Clinically, LCNEC should be differentiated from primary or metastatic carcinoid tumor, small cell carcinoma of pulmonary type and hypercalcemic type, undifferentiated ovarian carcinoma and metastatic neuroendocrine carcinoma (Table 3). Under light microscopy, the tumour comprised mostly of solid, sheets, islands, or bands patterns, occasionally with focal necrosis. The tumor cells were large and had large round to ovoid nuclei with coarse chromatin and numerous mitoses, typically have abundant eosinophilic cytoplasm that may be granular. It is difficult to diagnose LCNEC in ovary not only because of its clinical symptoms, CA125 level and lack of specificity in imaging, but also because of the atypical histological diagnosis of the disease. So it still requires the IHC to confirmed. Immunohistochemically the reported LCNEC were positive staining for neuroendocrine markers, such as NSE, chromogranin A, and synaptophysin, CD56, and various CKs.^[3]^ The presented case also showed positive immunoreactivity to CD56 and NSE but negative for the epithelial markers CK7 and CK20. Based on these, the present case fulfills the structure and immunohistochemical criteria for a primary pure ovarian LCNEC.

**Table 3:** Differential diagnosis and Pathological structure of of LCNEC.

Classification	Pathological structures and clinical abnormalities
Primary LCNEC	1. Solid sheets,islands,or bands patterns,large to intermediate in size, round to ovoid nuclei, coarse chromatin and numerous mitoses 2. Positive reactivity for CgA, Syn, CK and CD56
Primary or metastatic carcinoid tumor	Cytological consistency,with Low mitotic activity and absence of necrosis
Small cell carcinoma of pulmonary type	1. Smaller cell with obvious necrosis 2. Less intense immunohistochemical reaction for CK and CgA
Small cell carcinoma of hypercalcemic type	1. Hypercalcemia 2. large cells 3. Follicle-like spaces with pale intracytoplasmic hyaline globules
Undifferentiated ovarian carcinoma	1. Solid with hemorrhage and necrosis 2. Obvious cellular atypia and low differentiation 3. Immunohistochemical epithelial markers were positivity while neuroendocrine markers were negative
Metastatic neuroendocrine carcinoma	1. Bilateral ovarian involvement with multinodular growth pattern 2. Vascular invasion

CgA = chromogranin A, Syn = synaptophysin.

Ovarian LCNEC is a highly aggressive tumor with a poor prognosis even at an early stage. Similar to small cell carcinoma, irrespective of its stage, Ovarian LCNEC is very malignant and aggressive and recurrence or metastasis can occur within a short time in most patients.^[25]^ Due to the rarity of the disease and the lack of systematic population-based studies or registration data, there is hitherto no consensus on the optimal treatment. Clinically, surgical excision is mainly adopted and postoperative of platinum-based chemotherapy is supplemented. Radiotherapy may be an option for treatment.^[24]^ Oshita et al^[13]^ have reported a patient with brain metastasis and she had received wholebrain radiation therapy, and during the 64 months post initial treatment she did not experience tumor recurrence. However, the presented case has not received radiotherapy, and the effect is not clear. There is no consensus on standard treatment yet, but we believe that the combination of aggressive surgery with chemotherapy and adjuvant radiotherapy should be considered as a possible treatment strategy.

In conclusion, ovarian LCNEC is rare and defined as an extremely malignant tumor, with a poor prognosis even at an early stage. Early diagnosis is difficult, diagnosis requires histopathology and IHC. Due to the rarity of the disease and the lack of systematic population-based studies or registration data, there is hitherto no consensus on the optimal treatment.

## Author contributions

**Conceptualization:** Xue Peng, Hongjing Wang.

**Data curation:** Xue Peng.

**Investigation:** Xue Peng.

**Methodology:** Xue Peng.

**Validation:** Xue Peng.

**Writing – original draft:** Xue Peng.

**Writing – review & editing:** Xue Peng.
